# Nocturia, Sleep-Disordered Breathing, and Cardiovascular Morbidity in a Community-Based Cohort

**DOI:** 10.1371/journal.pone.0030969

**Published:** 2012-02-06

**Authors:** Sairam Parthasarathy, MaryPat Fitzgerald, James L. Goodwin, Mark Unruh, Stefano Guerra, Stuart F. Quan

**Affiliations:** 1 Department of Medicine, University of Arizona, Tucson, Arizona, United States of America; 2 Arizona Respiratory Center, University of Arizona, Tucson, Arizona, United States of America; 3 Department of Surgery, Hines Veterans Affairs Hospital, Hines, Illinois, United States of America; 4 Renal-Electrolyte Division, Department of Medicine, University of Pittsburgh, Pittsburgh, Pennsylvania, United States of America; 5 Center for Research in Environmental Epidemiology, Institute Municipal d'Investigació Mèdica, Consorcio de Investigación Biomédica de Epidemiología y Salud Pública(CIBERESP), Barcelona, Spain; 6 Division of Sleep Medicine, Harvard Medical School, Boston, Massachusetts, United States of America; Cardiff University, United Kingdom

## Abstract

**Background:**

Nocturia has been independently associated with cardiovascular morbidity and all-cause mortality, but such studies did not adjust for sleep-disordered breathing (SDB), which may have mediated such a relationship. Our aims were to determine whether an association between nocturia and cardiovascular morbidity exists that is independent of SDB. We also determined whether nocturia is independently associated with SDB.

**Methodology/Principal Findings:**

In order to accomplish these aims we performed a cross-sectional analysis of the Sleep Heart Health Study that contained information regarding SDB, nocturia, and cardiovascular morbidity in a middle-age to elderly community-based population. In 6342 participants (age 63±11 [SD] years, 53% women), after adjusting for known confounders such as age, body mass index, diuretic use, diabetes mellitus, alpha-blocker use, nocturia was independently associated with SDB (measured as Apnea Hypopnea index >15 per hour; OR 1.3; 95%CI, 1.2–1.5). After adjusting for SDB and other known confounders, nocturia was independently associated with prevalent hypertension (OR 1.23; 95%CI 1.08–1.40; P = 0.002), cardiovascular disease (OR 1.26; 95%CI 1.05–1.52; P = 0.02) and stroke (OR 1.62; 95%CI 1.14–2.30; P = 0.007). Moreover, nocturia was also associated with adverse objective alterations of sleep as measured by polysomnography and self-reported excessive daytime sleepiness (P<0.05).

**Conclusions/Significance:**

Nocturia is independently associated with sleep-disordered breathing. After adjusting for SDB, there remained an association between nocturia and cardiovascular morbidity. Such results support screening for SDB in patients with nocturia, but the mechanisms underlying the relationship between nocturia and cardiovascular morbidity requires further study. MeSH terms: Nocturia, sleep-disordered breathing, obstructive sleep apnea, sleep apnea, polysomnography, hypertension.

## Introduction

Sleep-disordered breathing (SDB) may lead to cardiovascular morbidity due to repetitive episodes of complete or partial upper airway obstruction [1], [2]. The mechanisms underlying such adverse cardiovascular effects are complex and are attributed, in part, to increased cardiac (stretch or) transmural pressure generated during occluded breathing, hypoxemia, and increased sympathetic activity (due to hypoxemia and arousals from sleep) [3]. Such pathophysiological mechanisms can also stimulate the secretion of natriuretic hormones [4] and thereby cause nocturia. Although previous case series and reports have reported nocturia as a manifestation of SDB [5], [6], [7], [8], [9], [10], a comprehensive and systematic study of the association between nocturia and SDB in a *large* community-based population has not been performed.

Moreover, nocturia *per se* may be associated with morbidity and mortality, rather than merely reflecting a consequence of adverse cardio-respiratory interactions [11]. Specifically, nocturia has been independently associated with falls, hypertension, and all-cause mortality in a study of elderly patients [12], [13], [14]. However, in such cohorts assessing the relationship between nocturia and hypertension, the investigators did not perform polysomnography nor did they adjust for SDB as a confounder [15], [16], [17], [18], [19]. Moreover, cohort studies have demonstrated an association between SDB and all cause mortality [20], [21], [22], [23], [24]. Hence, it is unclear as to whether nocturia is independently associated with hypertension, or whether such association may be attributable to residual confounding by co-existent SDB.

Our primary aim was to determine the cross-sectional associations between nocturia and cardiovascular morbidity exists (hypertension, coronary artery disease, heart failure, and stroke) that is independent of SDB in the Sleep Heart Health Study (SHHS) cohort. We hypothesized that self-reported frequency of nocturia is associated with prevalent hypertension and prevalent cardiovascular disease after adjusting for co-existent SDB. An additional aim was to determine whether nocturia is independently associated with SDB. We hypothesized that SDB is independently associated with self-reported frequency of nocturia in a large cohort of middle-aged and older individuals.

## Methods

### Study Population

This report uses data from the Sleep Heart Health Study (SHHS), a prospective multi-center cohort study that was performed to assess whether SDB is a risk factor for cardiovascular disease in middle-age and older adults. The SHHS had been approved by the institutional review board at each participating site. Informed written consent was obtained from all participants. Details regarding the design and methodology are available elsewhere [25], [26]. Briefly, participants were recruited from ongoing cohort studies, that included the Cardiovascular Health Study [27], the Framingham Heart Study [28], the Tucson Epidemiologic Study of Obstructive Airways Disease [29], the Strong Heart Study [30], the Atherosclerosis Risk in Communities Study [31], the Health and Environment Cohort Study in Tucson, as well as three New York City cohorts undergoing evaluation for the impact of psychosocial risk factors on cardiovascular disease. Inclusion criteria included age ≥40 years and not having received positive airway pressure treatment for sleep apnea, a tracheostomy, or supplemental oxygen. Participants underwent unattended PSG at home between December 1995 and January 1998.

### Polysomnography

Polysomnography (PSG) was performed in an unattended setting at home (Compumedics PS-2 system; Compumedics Pty. Ltd, Abbotsville, Australia). Recordings included electroencephalogram (C3/A1 and C4/A2), right and left electrooculograms, submental electromyogram, nasal/oral airflow recorded by thermocouple (Protech, Woodenville, WA), rib cage and abdominal movement recorded by inductive plethysmography, oxyhemoglobin saturation (SpO_2_) by pulse oximetry (Nonin, Minneapolis, MN), and electrocardiogram. Leg movements were not recorded. Standardized techniques for sensor attachment and quality assurance were used and have been previously described [25].

### PSG Scoring and Sleep Parameters

Scoring of sleep stages followed the guidelines of Rechtschaffen and Kales and were performed at a centralized location [32]. Apnea was defined as complete or almost complete cessation of airflow (<25% of baseline) and associated with 4% desaturation or more. Hypopnea was defined as a decrease to less than 70% of baseline on either inductance plethysmography channel or thermocouple channels, for 10 seconds or longer and associated with 4% desaturation or more. The apnea hypopnea index (AHI) was calculated by computing the average number of apneas plus hypopneas per hour of sleep.

### Health and Demographic Data

Demographic information was obtained from the parent cohorts. Participants' weight was measured using a calibrated scale at the time of PSG. Participants' height was taken from parent study data obtained within 3 years of the sleep study and used to calculate the body mass index (BMI) (kg/m^2^). Health information was derived from several sources. Data related to chronic medical conditions (such as diabetes mellitus) was obtained from the parent cohorts. Current medications were recorded on the night of the PSG. The SHHS Sleep Habits Questionnaire incorporated the Epworth Sleepiness Scale.

An interviewer administered health interview on the night of the PSG was used to obtain information pertaining to self-reported prevalent cardiovascular disease, smoking and caffeine use. In the Sleep Heart Health Study (SHHS), the interviewer asked if a doctor had ever told the participant that she or he had angina, heart attack, heart failure, or stroke and if the participant ever underwent coronary artery bypass surgery or coronary angioplasty. An “unsure” response was allowed for each item. We then defined prevalent cardiovascular disease as a positive response to one or more of the afore-mentioned conditions or procedures. Prevalent coronary heart disease was similarly defined, excluding the questions about heart failure (which sometimes has a coronary etiology) and stroke. Those who responded “No” to all of the questions were considered free of cardiovascular disease. The remainder (i.e., a combination of “no” and “Unsure” responses to all of the items) were classified as unknown disease status and were considered missing in these analyses. Data pertaining to certain covariates such as chronic medical conditions (diabetes mellitus) was collected from the parent cohort as long as such information was collected within a 3 month window of the home sleep study. If such covariate information pertaining to chronic medical conditions was collected outside the 3-month window, then such variables had to be re-ascertained during the interview. Hypertension was considered present if the participant had a blood pressure of at least 140/90 mm Hg on the night of the PSG, or was currently being treated with antihypertensive medications.

### Categorization and codification of nocturia variable

Data on nocturnal urinary frequency was obtained from the SHHS Sleep Habits Questionnaire. Participants were queried as to how often they awakened to go to the bathroom: never, rarely (1/month or less), sometimes (2–4/month), often (5–15/month), and almost always (16–30/month). For the purpose of analysis in this study, nocturia was analyzed both as a continuous variable as well as a categorical variable after collapsing the groups. For the continuous variable, we codified the five ordinal categories of nocturia in the following manner: never = 1; rarely = 2, sometimes = 3; often = 4; and almost always = 5. Also, we collapsed this ordinal ‘nocturia’ variable into a dichotomous categorical variable (nocturia present or absent) by progressively increasing the threshold level of collapse from ‘rarely’ to ‘often’ to yield 4 different dichotomous variables for nocturia. Our rationale for such analytical methodology was the following: first, nocturia is *not usually* defined by a particular threshold volume or frequency of nocturia; second, by international consensus, nocturia is simply defined as waking at night to void, which implies that nocturia is a continuum from normal to a bothersome or morbidity-associated abnormal condition [33]. We subsequently used the dichotomous variable that was most strongly associated with SDB for further analysis.

### Statistical Analyses

Proportions were analyzed by χ^2^, and continuous variables were compared by unpaired t-tests or the non-parametric equivalent (Mann-Whitney) test. Logistic regression was used to calculate the odds ratio (OR) of nocturia while comparing SDB categories and adjusting for possible confounders. In these analyses, AHI was categorized by commonly used clinical cutoff points (5, 10, and 15 per hour). Univariate and multivariate linear regressions with nocturia expressed as a dependent linear variable was performed in order to determine whether nocturia was associated with SDB. Binary logistic regression was performed to identify significant variables and all variables significant at P<0.05 level were included as independent variables in multivariate logistic regression models (forced entry). All analyses were performed using SPSS for Windows Version 12.0.1. Data are presented as mean ± SD or median and interquartile range wherever appropriate.

## Results

**Figure 1:**
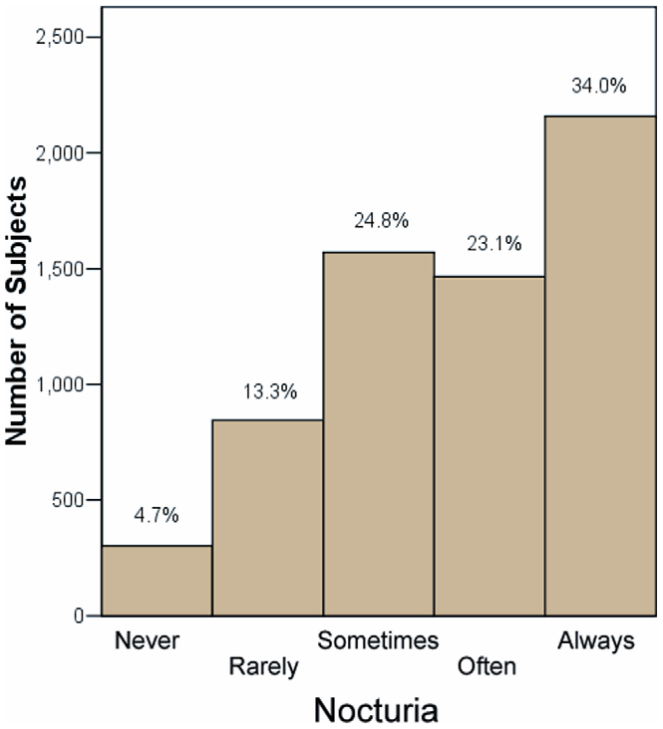
Proportions and numbers of patients with reported frequency of nocturia. Participants were queried as to how often in the prior year, did they awaken to go to the bathroom: never, rarely (1/month or less), sometimes (2–4/month), often (5–15/month), and almost always (16–30/month).

**Figure 2:**
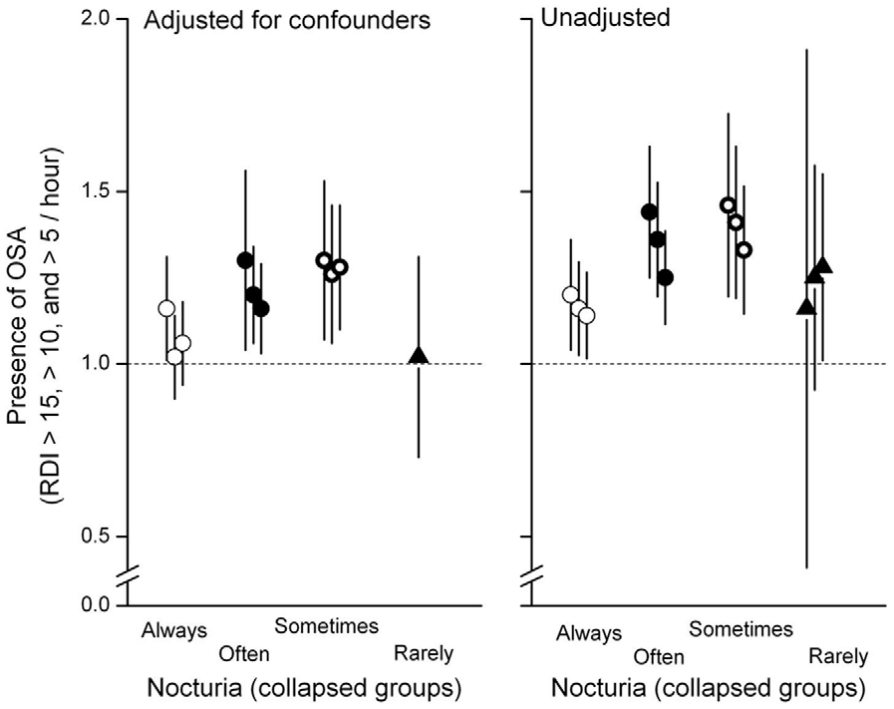
Association between nocturia and presence of sleep-disordered breathing based upon different apnea-hypopnea index (AHI) thresholds are shown as odds ratio (*symbol*) and 95% confidence intervals (*y-error bars*). Odds ratios and respective confidence intervals that were adjusted for confounders (*left panel*) and unadjusted for confounders (*right panel*) are shown. The x-axis of each panel represents nocturia expressed as a dichotomous categorical variable (nocturia present or absent) by progressively increasing the threshold level of collapse from ‘rarely’ to ‘always’ to yield four different dichotomous variables for nocturia. The cluster of three symbols with corresponding error bars for each definition of nocturia correspond to variable AHI thresholds for nocturia from left-to-right (>15, >10 and >5 per hour, respectively). For nocturia defined as greater than rarely (*closed triangle*) only one adjusted odds ratio is shown (*left panel*) because multivariate regression was performed only if univariate regression was significant at P<0.05 (see methods).

The characteristics of the patients with and without nocturia are shown in **Table 1**. Patients with nocturia were older, had greater body mass index (BMI), and were more likely to be receiving diuretic agents or alpha-blockers (likely for prostatism). In 6342 participants (age 63±11 [SD] years, 53% women) self-reported levels of nocturia are depicted in **Figure 1** **.** In **Table 2**, increasing severity of nocturia – expressed as an ordinal variable – was associated with increasing severity of SDB, increasing age, increasing BMI, greater alcohol consumption, diuretic intake, presence of diabetes mellitus, lower lung function (measured as forced expiratory volume in 1 second [FEV_1_]), greater sleep fragmentation (time awake after sleep onset and arousals per hour of sleep) and α-blocker ingestion. After adjusting for such confounders, the association between nocturia and SDB remained (**Table 2**). Collapsing the ordinal variable differently – in order to identify the threshold level of nocturia frequency that was most strongly associated with SDB – yielded two threshold levels (at least 5 nights per month or at least 16 nights per month) that were significantly associated with SDB (**Figure 2** **)**. Using the former definition (at least 5 nights per month), 3625 (57.1%) patients were classified as having nocturia (**Figure 1**).

**Table 1:** Participant Characteristics.

Variable	Nocturia	No nocturia	P value
Age (years)	64.2±10.5	61.2±11.3	<0.0001
Men (proportion)	56.7%	57.6%	0.49
Body Mass Index (Kg/m^2^)	28.7±5.5	28.3±5.2	0.007
FEV1 (Liters)	2.62±0.80	2.77±3.3	0.02
Alcohol consumption (drinks per night)	0 (0, 3)	0 (0, 4)	0.49
Diuretic intake (proportion)	17.0%	13.4%	<0.0001
Diabetes Mellitus (proportion)	11.4%	10.0%	0.08
Alpha blockers (proportion)	6.1%	2.8%	<0.0001
AHI (4% hypopnea)	4.6 (1.6, 11.1)	5.7 (1.9, 13.9)	<0.0001
Patients with AHI >15 events/hr	23.2%	17.4%	<0.0001
Patients with AHI >10 events/hr	33.9%	27.3%	<0.0001
Patients with AHI >5 events/hr	39.8%	48.2%	<0.0001

Kg/m^2^ = kilogram/meter^2^; FEV1 = Forced expiratory volume; hr = hour; proportions compared by Chi square; Mann-Whitney test for non-parametrics (presented as median and interquartile range); unpaired t-test for parametric variables (presented as mean ± SD); AHI = apnea-hypopnea index.

**Table 2:** Variables Associated With Presence Of Nocturia.†

Variable	B (S.E.)	P value	95% CI
AHI†	0.008 (0.001)	<0.0001	0.005, 0.01
AHI >15*	0.18 (0.04)	<0.0001	0.12, 0.27
AHI >10*	0.17 (0.04)	<0.0001	0.10, 0.24
AHI >5*	0.14 (0.03)	<0.0001	0.07, 0.20
Age	0.015 (0.001)	<0.0001	0.013, 0.018
Body mass index	0.009 (0.003)	0.002	0.003, 0.014
Alcohol	0.005 (0.002)	0.046	0, 0.01
Diuretics*	0.14 (0.04)	0.001	0.05, 0.22
Diabetes Mellitus*	0.11 (0.05)	0.034	0.008, 0.20
α-blocker*	0.51 (0.07)	<0.001	0.37, 0.65
FEV_1_	−0.20 (0.01)	0.003	−0.03, −0.01
Coffee	−0.03	<0.0001	−0.04, −0.02
Soda	−0.06	<0.0001	−0.09, −0.03
Tea	0.01	0.4	−0.013, 0.04
**Multiple regressions** §			
AHI†	0.004 (0.001)	0.001	0.002, 0.007
AHI >15*	0.12 (0.04)	0.007	0.03, 0.21
AHI >10*	0.07 (0.04)	0.06	−0.01, 0.15
AHI >5*	0.07 (0.04)	0.04	0.0, 0.14

AHI = respiratory disturbance index; BMI = body mass index; FEV_1_ = forced expiratory volume in one second; CI = confidence interval;

*dichotomous variables;

† continuous variable.

§ Multiple regression that adjusts for age, BMI, alcohol, diuretics, FEV_1_, α-blocker, coffee and soda.

### Relationship between nocturia and SDB

Univariate logistic regression identified the following covariates associated with nocturia: greater age, higher body mass index, lower forced expiratory volume in one second, ingestion of any diuretic, ingestion of alpha blocker agents, and the presence of SDB based on a thresholds of 5, 10, or 15 events per hour for the AHI (P<0.05)(**Table 3**). Presence of diabetes mellitus and ingestion of alcohol also had a tendency to be associated with nocturia (P = 0.08; **Table 3**).

**Table 3:** Unadjusted And Adjusted Odds Ratios Of Variables Associated With Nocturia.∥∥

Variable	Unadjusted Odds Ratio (95% CI)
**Univariate regressions**	
Age*	1.03 (1.02–1.03)†††
Gender†	0.97 (0.87–1.07)
Body mass index‡	1.01 (1.00–1.02)***
Diabetes Mellitus§	1.16 (0.98–1.37)
Diuretic∥	1.33 (1.16–1.53)†††
Alcohol use**	1.008 (0.99–1.02)
Alpha blocker use††	2.25(1.72–2.93)†††
Coffee intake (cups per day)	0.95 (0.93, 0.97)†††
Tea intake (cups per day)	1.01 (0.97, 1.05)
Soda intake (cans per day)	0.91 (0.87, 0.95)†††
FEV1‡‡	0.88 (0.83–0.94)†††
AHI >15 ^§§^	1.44 (1.26–1.65)†††
AHI >10 ^§§^	1.36 (1.21–1.53)†††
AHI >5 ^§§^	1.25 (1.12–1.39)†††
**Multivariate regressions** @	**Adjusted odds ratios**
AHI >15 ^§§^	1.31 (1.13–1.51)†††
AHI >10 ^§§^	1.2 (1.06–1.36)***
AHI >5 ^§§^	1.16 (1.03–1.30)***

@ Adjusted for age, body mass index, diuretics, alpha-blockers, and FEV1. FEV1 = Forced expiratory volume in 1 second; AHI = apnea hypopnea index; B = estimated coefficient; S.E. = standard error; CI = confidence intervals;

*per unit year age;

† compared to women;

‡ per unit Kg/m^2^;

§ compared to no history of diabetes mellitus;

∥ compared to no diuretic intake;

**continuous variable (number of drinks per day);

†† compared to no alpha-blocker use;

‡‡ per liter FEV1;

∥∥ dichotomous variable. Nocturia was defined as ‘often’ (occurring at least 5 times per month).

***P<0.05;

††† P<0.0001.

Multiple logistic regression, with nocturia as the dependent variable, revealed that after adjusting for age, body mass index, ingestion of diuretics, alcohol consumption, diabetes mellitus, and alpha blocker agents, and FEV_1_, the presence of SDB was independently associated with nocturia using all 3 cutoffs for AHI (5, 10, or 15 per hour) with hypopneas requiring >4% oxygen desaturation. However, statistical significance was not achieved for AHI >5 per hour (**Table 3**).

### Relationship between nocturia, hypertension, and cardiovascular disease

Univariate logistic regression identified the following covariates were associated with hypertension: nocturia, greater severity of SDB (AHI with 4% oxygen desaturation; continuous variable), greater age, higher body mass index, ethnicity, cigarette smoking, time awake after sleep onset, arousals per hour of sleep, alcohol ingestion, neck circumference, and waist-hip ratio (**Table 4**). Using multiple logistic regression, nocturia was independently associated with hypertension after adjusting for age, body mass index, ethnicity, cigarette smoking, alcohol ingestion, neck circumference, and waist-hip ratio, diuretics and severity of SDB (AHI with hypopneas requiring at least 4% oxygen desaturation; (**Table 4**).

**Table 4:** Adjusted And Unadjusted Odds Ratios Of Associations with Hypertension.

Variable	Unadjusted Odds ratio(95% CI)
**Univariate regressions**	
Nocturia	1.44 (1.30–1.59)†††
Age	1.05 (1.05–1.06)†††
Body mass index	1.04 (1.03–1.05)†††
African-Americans*	0.55 (0.46–0.66)†††
Gender	1.08 (0.98–1.19)
Cigarette pack years†	1.004 (1.002–1.006)†††
Alcohol	0.99 (0.99–1.00)
Neck circumference‡	1.06 (1.05–1.07)†††
Diabetes Mellitus§	2.4 (2.04–2.84)†††
AHI∥	1.02 (1.02–1.03)†††
Diuretics**	96.7 (62.5–149.6)†††
Waist-hip ratio††	8.49 (4.78–15.1)†††
**Multivariate Regressions with variables adjusted for:**	**Adjusted Odds Ratios**
**Diuretics, age, BMI, ethnicity, smoking, neck size, diabetes, waist-hip ratio (Model r^2^ = 0.38)**	
Nocturia‡‡	1.23 (1.08–1.40)***
**AHI, diuretics, age, BMI, ethnicity, smoking, neck size, diabetes, waist-hip ratio (Model r^2^ = 0.38)**	
Nocturia‡‡	1.22 (1.06–1.40)***
**AHI, diuretics, age, BMI, ethnicity, smoking, neck size, diabetes, waist-hip ratio, WASO** §§ **(Model r^2^ = 0.39)**	
Nocturia‡‡	1.20 (1.04–1.38)***
**AHI, diuretics, age, BMI, ethnicity, smoking, neck size, diabetes, waist-hip ratio, Arousals** ∥∥ **(Model r^2^ = 0.39)**	
Nocturia‡‡	1.22 (1.06–1.39)***

BMI = body mass index; AHI = apnea hypopnea index; diabetes = diabetes mellitus; B = estimated coefficient; SE = standard error; CI = confidence intervals.

*African-Americans compared to all other races combined;

† per cigarette pack year;

‡ per inch neck circumference;

§ compared to absence of diabetes mellitus;

∥ per unit apnea-hypopnea index (>4% desaturation for hypopnea; *continuous variable*);

**compared to no diuretic;

†† per unit change in ratio.

§§ WASO = time awake after sleep onset;

∥∥ Arousals = arousal index expressed as arousals per hour of sleep;

‡‡ Nocturia was defined as ‘often’ (occurring at least 5 times per month).

***P<0.05;

††† P<0.0001.

Nocturia was independently associated with cardiovascular disease and stroke after adjusting for SDB and other known confounders for cardiovascular disease, whereas, nocturia was associated with a reduced odds ratio for presence of heart failure (**Table 5**). History of coronary artery disease was not associated with presence of nocturia (**table 5**).

**Table 5:** Adjusted Odds Ratios Of Associations Between Nocturia and Prevalent Cardiovascular Morbidity.

Variable	95% CI
**Cardiovascular disease**	
Adjusted for cardiovascular risk factors*	1.23 (1.03–1.46)‡
Adjusted for cardiovascular risk factors and SDB†	1.26 (1.05–1.52)‡
Adjusted for cardiovascular risk factors, SDB, WASO§	1.26 (1.05–1.53)‡
Adjusted for cardiovascular risk factors, SDB, arousals∥	1.28 (1.05–1.54)‡
**Coronary artery disease**	
Adjusted for cardiovascular risk factors*	1.14 (0.95–1.37)
Adjusted for cardiovascular risk factors and SDB†	1.12 (0.92–1.36)
Adjusted for cardiovascular risk factors, SDB, WASO§	1.12 (0.92–1.36)
Adjusted for cardiovascular risk factors, SDB, arousals∥	1.14 (0.93–1.39)
**Heart failure**	
Adjusted for cardiovascular risk factors*	0.72 (0.48–1.08)
Adjusted for cardiovascular risk factors and SDB†	0.59 (0.38–0.92)‡
Adjusted for cardiovascular risk factors, SDB, WASO§	0.58 (0.37–0.91)‡
Adjusted for cardiovascular risk factors, SDB, arousals∥	0.57 (0.36–0.90)‡
**Stroke**	
Adjusted for cardiovascular risk factors*	1.39 (1.01–1.91)‡
Adjusted for cardiovascular risk factors and SDB†	1.62 (1.14–2.30)‡
Adjusted for cardiovascular risk factors, SDB, WASO§	1.61 (1.13–2.29)‡
Adjusted for cardiovascular risk factors, SDB, arousals∥	1.67 (1.16–2.40)‡

*Adjusted for age, gender, race, smoking, diabetes mellitus, hypertension, systolic blood pressure, body mass index, total cholesterol, and high density lipoprotein levels.

† Adjusted for sleep-disordered breathing (SDB; measured as apnea-hypopnea index) in addition to other confounders listed^*^. Nocturia was defined as ‘often’ (occurring at least 5 times per month).

§ WASO = time awake after sleep onset;

∥ arousal index expressed as arousals per hour of sleep;

‡ P<0.05.

### Polysomnographic changes and Sleepiness

Patients with nocturia displayed greater derangement of polysomnographic measures than patients without nocturia (**Table 6**). Those with nocturia had less total sleep time, sleep efficiency and proportion of REM sleep, but had a higher arousal index and were more hypoxemic. They also were subjectively more sleepy on the basis of their Epworth Sleepiness Score.

**Table 6:** Polysomnographic variables and subjective sleepiness.

PolysomnographyVariable	Nocturia	NoNocturia	P value
Sleep Latency (min)*	16.5 (9.5, 28.0)	16.5 (9.5, 29.5)	0.52
Sleep time (min)	365 (317, 404)	367 (322, 408)	0.06
Total sleep period (min)	448 (405, 482)	440 (396, 477)	<0.0001
Sleep efficiency (%)*	82.8 (75.4, 88.0)	85.1 (77.4, 90.1)	<0.0001
Wake after sleep onset (min)	55.5 (34.0, 87.0)	43.5 (26.5, 76.5)	<0.0001
NREM Stage 1 (%)	4.6 (2.8, 7.2)	4.5 (2.8, 7.1)	0.32
NREM Stage 2 (%)	57.5 (49.3, 65.4)	57.2 (49.3, 64.9)	0.30
NREM Stage 3 & 4 (%)	16.7 (8.2, 25.7)	17.0 (8.2, 24.6)	0.36
REM (%)	19.8 (15.4, 23.7)	20.5 (16.5, 24.3)	<0.0001
REM latency (min)	75.0 (56.5, 111.0)	72.5 (56.0, 100.5)	0.006
Arousal Index	17.3 (12.4, 24.3)	16.2 (11.6, 23.0)	<0.0001
Time spent SpO2 <90%	0.28 (0.01, 2.35)	0.17 (0.0, 1.55)	<0.0001
Time spent SpO2 <85%	0.0 (0.0, 0.08)	0.0 (0.0, 0.03)	<0.0001
Epworth sleepiness score	8 (5, 11)	6 (4, 10)	<0.0001

Min = minutes, % = percentage; NREM = non-rapid eye movement sleep; REM = rapid eye movement sleep; SpO2 = oxygen saturation by pulseoximetry;

*less than full compliments of sample size (3688 to 5837). Nocturia was defined as ‘often’ (occurring at least 5 times per month).

## Discussion

Nocturia was independently associated with SDB in a large community-based sample of middle-aged and older subjects. Although previous reports had identified nocturia as a manifestation of SDB, such studies had used either a much smaller sample size (ranging from 5 to 138 subjects) [5], [6], [7], [8], [9], [34], [35] or had studied a sleep lab-based population [6], [7], [8], [9], [10], [34], [35]. Our study findings are therefore more generalizable as we studied a large community-based sample of middle-aged to older population. Moreover, our cross-sectional study revealed a robust relationship between nocturia and SDB after adjustment for many potential confounders. The association with SDB has important implications to patient care and research. Primary care physicians and urologists should consider SDB in the differential diagnosis of patients who present with nocturia. In addition, the mechanistic underpinning for the relationship between nocturia and SDB, viz., natriuretic peptides and the stimulus for release of such natriuretic peptides by the adverse cardio-respiratory effects of SDB (hypoxia, increased respiratory effort [increased transmural pressure], and arousals from sleep) and rostral fluid shifts may be worthy of further research [36].

In our study, we also show that nocturia was independently associated with prevalent hypertension, cardiovascular disease, and stroke. Such an association was independent of co-existent SDB measured by the AHI (**Tables 4** **and** **5**). Previous studies have demonstrated an association between nocturia and cardiovascular morbidity, however, none of these studies measured or adjusted for co-existent SDB [12], [15], [16], [17], [18], [19]. Therefore, prior to this report, whether nocturia was associated with hypertension independent of co-existent SDB was unclear.

The association between nocturia and cardiovascular morbidity raises some intriguing questions. First, what is the mechanism underlying the association between nocturia and cardiovascular morbidity? When considering this question, we should note that the association between hypertension and nocturia was also independent of the effects of diuretics (**Table 4**). One potential postulated mechanism for the association between nocturia and cardiovascular morbidity has been the adverse hemodynamic changes associated with the stress of nocturnal awakenings [37]. It is conceivable that nocturia, by virtue of disrupting sleep and causing insufficient sleep, may lead to adverse cardiovascular effects. There is an evolving, large body of literature concerning the effects of poor or insufficient sleep on increased blood pressure, glucose intolerance, and inflammation, which, may in turn, lead to adverse cardiovascular morbidity [38]. In our report, however, while sleep duration and efficiency were statistically lower in subjects experiencing nocturia (when compared to subjects without nocturia) the difference was small (**Table 6**). Such data would suggest that mechanisms other than insufficient sleep may be responsible for the association between nocturia and cardiovascular morbidity. Moreover, the multivariate regressions revealed that nocturia was associated with cardiovascular disease independent of sleep fragmentation measured as time awake after sleep onset or arousals per hour of sleep (**tables 4** **and** **5**). Such findings would suggest that the association between nocturia and cardiovascular disease is independent of known associations between adequate sleep and cardiovascular disease [39].

Second, different cardiovascular morbidities expressed divergent relationships with the presence of nocturia. Nocturia was associated with an increased risk for hypertension, cardiovascular disease, and stroke, whereas, presence of nocturia appeared to be protective against heart failure (**Table 5**). Conceivably, nocturia could reduce circulating blood volume and reduce cardiac preload in patients with heart failure. However, such reasoning would be at odds with data suggesting that the up-regulation of the neurohormonal process underlying generation of nocturia – natriuretic peptides – is associated with increased cardiovascular mortality [40], [41]. Such data may in turn be reconciled by evidence suggesting that certain polymorphisms in natriuretic peptides may be associated with reduced bioactivity of such natriuretic hormones and consequently can influence outcomes in heart failure [42]. We realize that the effects of residual confounding cannot be excluded as a potential cause for such an association. Clearly, the relationship between natriuretic peptides, nocturia, and cardiovascular outcomes is complex and needs more research.

The findings of this report should be considered in light of a number of limitations. First, the association between nocturia and SDB does not necessarily imply a cause-effect relationship based on our data. However, other studies have demonstrated the improvement of nocturia following continuous positive airway pressure therapy and can attribute nocturia – at least in part – to SDB [34], [35], [43]. Second, our study did not consider concomitant measures relevant to nocturia such as daily water intake or diurnal variation in urine production. Conceivably, greater ingestion of fluids in patients with SDB who suffer from dry throat sensation may play a role in the observed association between nocturia and SDB. Third, we used AHI as the primary measure of the insults imposed by SDB and did not consider a composite index that included hypoxia and arousals – both mechanistically implicated in the causation of nocturia by SDB. However, arousals alone have not always been shown to be closely associated with cardiovascular morbidity. Fourth, our report could have been strengthened by accounting for the confounding influence of kidney disease, which, through the loss of renal concentrating ability may lead to polyuria and accompanying nocturia. However, in the SHHS study, while the individual sub-cohorts had different measures relating to kidney function that were obtained at different points in time, there was not a uniform measure of kidney function for the entire SHHS cohort obtained at the same time relative to the SHHS protocol. We did find that measures of hypoxemia (number of oxygen desaturations per hour and time spent below 90%) were indeed associated with nocturia (data not shown). However, as anticipated, such hypoxemia variables and the AHI were closely correlated. Therefore we chose to only use the measure with the stronger association with nocturia in our regression models – viz., apnea-hypopnea index. Lastly, the study variables regarding nocturia and cardiovascular disease were based upon self-reported cross-sectional data obtained at enrollment. Nocturia was not measured from urine volume and the presence of cardiovascular disease (other than hypertension) was not cross-referenced against patient medical records. However, because of the difficulty in measuring overnight nocturnal urine production, nocturia has been defined quite simply by international consensus as just waking at night to void [33].

In conclusion, nocturia is independently associated with sleep-disordered breathing. After adjusting for SDB, there remained an association between nocturia and cardiovascular morbidity. Such results support screening for SDB in patients with nocturia, but the mechanisms underlying the relationship between nocturia and cardiovascular morbidity require further study. These data suggest that clinicians should be cognizant of the association between nocturia and SDB, and consider screening for SDB in patients with recalcitrant nocturia.

## References

[1] Nieto FJ, Young TB, Lind BK, Shahar E, Samet JM (2000). Association of sleep-disordered breathing, sleep apnea, and hypertension in a large community-based study. Sleep Heart Health Study.. JAMA.

[2] O'Connor GT, Caffo B, Newman AB, Quan SF, Rapoport DM (2009). Prospective study of sleep-disordered breathing and hypertension: the Sleep Heart Health Study.. Am J Respir Crit Care Med.

[3] Leung R, Bradley TD (2001). Sleep apnea and cardiovascular disease.. Am J Respir Crit Care Med.

[4] de Lemos JA, McGuire DK, Drazner MH (2003). B-type natriuretic peptide in cardiovascular disease.. Lancet.

[5] Chasens ER, Umlauf MG, Pillion DJ, Wells JA (2002). Nocturnal polyuria in type 2 diabetes: a symptom of obstructive sleep apnea.. Diabetes Educ.

[6] Hajduk IA, Strollo PJ, Jasani RR, Atwood CW, Houck PR (2003). Prevalence and predictors of nocturia in obstructive sleep apnea-hypopnea syndrome–a retrospective study.. Sleep.

[7] Kiely JL, Murphy M, McNicholas WT (1999). Subjective efficacy of nasal CPAP therapy in obstructive sleep apnoea syndrome: a prospective controlled study.. Eur Respir J.

[8] Kramer NR, Bonitati AE, Millman RP (1998). Enuresis and obstructive sleep apnea in adults.. Chest.

[9] Pressman MR, Figueroa WG, Kendrick-Mohamed J, Greenspon LW, Peterson DD (1996). Nocturia. A rarely recognized symptom of sleep apnea and other occult sleep disorders.. Arch Intern Med.

[10] Romero E, Krakow B, Haynes P, Ulibarri V (2009). Nocturia and snoring: predictive symptoms for obstructive sleep apnea.. Sleep Breath.

[11] Kupelian V, Fitzgerald MP, Kaplan SA, Norgaard JP, Chiu GR (2011). Association of nocturia and mortality: results from the Third National Health and Nutrition Examination Survey.. J Urol.

[12] Stewart RB, Moore MT, May FE, Marks RG, Hale WE (1992). Nocturia: a risk factor for falls in the elderly.. J Am Geriatr Soc.

[13] Asplund R (1999). Mortality in the elderly in relation to nocturnal micturition.. BJU Int.

[14] Johnson TM, Sattin RW, Parmelee P, Fultz NH, Ouslander JG (2005). Evaluating potentially modifiable risk factors for prevalent and incident nocturia in older adults.. J Am Geriatr Soc.

[15] Yoshimura K, Terada N, Matsui Y, Terai A, Kinukawa N (2004). Prevalence of and risk factors for nocturia: Analysis of a health screening program.. Int J Urol.

[16] Kupelian V, Rosen RC, Link CL, McVary KT, Aiyer LP (2009). Association of urological symptoms and chronic illness in men and women: contributions of symptom severity and duration–results from the BACH Survey.. J Urol.

[17] Bing MH, Moller LA, Jennum P, Mortensen S, Lose G (2008). Nocturia and associated morbidity in a Danish population of men and women aged 60–80 years.. BJU Int.

[18] Hsieh CH, Kuo TC, Hsu CS, Chang ST, Lee MC (2008). Nocturia among women aged 60 or older in Taiwan.. Aust N Z J Obstet Gynaecol.

[19] Fitzgerald MP, Litman HJ, Link CL, McKinlay JB (2007). The association of nocturia with cardiac disease, diabetes, body mass index, age and diuretic use: results from the BACH survey.. J Urol.

[20] Marshall NS, Wong KK, Liu PY, Cullen SR, Knuiman MW (2008). Sleep apnea as an independent risk factor for all-cause mortality: the Busselton Health Study.. Sleep.

[21] Punjabi NM, Caffo BS, Goodwin JL, Gottlieb DJ, Newman AB (2009). Sleep-disordered breathing and mortality: a prospective cohort study.. PLoS Med.

[22] Marin JM, Carrizo SJ, Vicente E, Agusti AG (2005). Long-term cardiovascular outcomes in men with obstructive sleep apnoea-hypopnoea with or without treatment with continuous positive airway pressure: an observational study.. Lancet.

[23] Young T, Finn L, Peppard PE, Szklo-Coxe M, Austin D (2008). Sleep disordered breathing and mortality: eighteen-year follow-up of the Wisconsin sleep cohort.. Sleep.

[24] Gooneratne NS, Richards KC, Joffe M, Lam RW, Pack F (2011). Sleep disordered breathing with excessive daytime sleepiness is a risk factor for mortality in older adults.. Sleep.

[25] Quan SF, Howard BV, Iber C, Kiley JP, Nieto FJ (1997). The Sleep Heart Health Study: design, rationale, and methods.. Sleep.

[26] Redline S, Sanders MH, Lind BK, Quan SF, Iber C (1998). Methods for obtaining and analyzing unattended polysomnography data for a multicenter study. Sleep Heart Health Research Group.. Sleep.

[27] Fried LP, Borhani NO, Enright P, Furberg CD, Gardin JM (1991). The Cardiovascular Health Study: design and rationale.. Ann Epidemiol.

[28] Dawber TR, Kannel WB, Lyell LP (1963). An approach to longitudinal studies in a community: the Framingham Study.. Ann N Y Acad Sci.

[29] Lebowitz MD, Knudson RJ, Burrows B (1975). Tucson epidemiologic study of obstructive lung diseases. I: Methodology and prevalence of disease.. Am J Epidemiol.

[30] Lee ET, Welty TK, Fabsitz R, Cowan LD, Le NA (1990). The Strong Heart Study. A study of cardiovascular disease in American Indians: design and methods.. Am J Epidemiol.

[31] (1989). The Atherosclerosis Risk in Communities (ARIC) Study: design and objectives. The ARIC investigators.. Am J Epidemiol.

[32] Rechtschaffen AKA (1968). A manual of standardized terminology, techniques and scoring system for sleep stages of human subjects..

[33] Van Kerrebroeck P, Abrams P, Chaikin D, Donovan J, Fonda D (2002). The standardization of terminology in nocturia: report from the standardization subcommittee of the International Continence Society.. BJU Int.

[34] Fitzgerald MP, Mulligan M, Parthasarathy S (2006). Nocturic frequency is related to severity of obstructive sleep apnea, improves with continuous positive airways treatment.. Am J Obstet Gynecol.

[35] Guilleminault C, Lin CM, Goncalves MA, Ramos E (2004). A prospective study of nocturia and the quality of life of elderly patients with obstructive sleep apnea or sleep onset insomnia.. J Psychosom Res.

[36] Friedman O, Bradley TD, Chan CT, Parkes R, Logan AG (2010). Relationship between overnight rostral fluid shift and obstructive sleep apnea in drug-resistant hypertension.. Hypertension.

[37] Bursztyn M, Jacob J, Stessman J (2006). Usefulness of nocturia as a mortality risk factor for coronary heart disease among persons born in 1920 or 1921.. Am J Cardiol.

[38] Mullington JM, Haack M, Toth M, Serrador JM, Meier-Ewert HK (2009). Cardiovascular, inflammatory, and metabolic consequences of sleep deprivation.. Prog Cardiovasc Dis.

[39] Ayas NT, White DP, Manson JE, Stampfer MJ, Speizer FE (2003). A prospective study of sleep duration and coronary heart disease in women.. Arch Intern Med.

[40] Zethelius B, Berglund L, Sundstrom J, Ingelsson E, Basu S (2008). Use of multiple biomarkers to improve the prediction of death from cardiovascular causes.. N Engl J Med.

[41] Kragelund C, Gronning B, Kober L, Hildebrandt P, Steffensen R (2005). N-terminal pro-B-type natriuretic peptide and long-term mortality in stable coronary heart disease.. N Engl J Med.

[42] Vassalle C, Andreassi MG, Prontera C, Fontana M, Zyw L (2007). Influence of ScaI and natriuretic peptide (NP) clearance receptor polymorphisms of the NP System on NP concentration in chronic heart failure.. Clin Chem.

[43] Margel D, Shochat T, Getzler O, Livne PM, Pillar G (2006). Continuous positive airway pressure reduces nocturia in patients with obstructive sleep apnea.. Urology.

